# promor: a comprehensive R package for label-free proteomics data analysis and predictive modeling

**DOI:** 10.1093/bioadv/vbad025

**Published:** 2023-03-07

**Authors:** Chathurani Ranathunge, Sagar S Patel, Lubna Pinky, Vanessa L Correll, Shimin Chen, O John Semmes, Robert K Armstrong, C Donald Combs, Julius O Nyalwidhe

**Affiliations:** Eastern Virginia Medical School, School of Health Professions, Norfolk, VA 23501, USA; Eastern Virginia Medical School, School of Health Professions, Norfolk, VA 23501, USA; Eastern Virginia Medical School, School of Health Professions, Norfolk, VA 23501, USA; The Leroy T. Canoles Jr. Cancer Research Center, Eastern Virginia Medical School, Norfolk, VA 23501, USA; The Leroy T. Canoles Jr. Cancer Research Center, Eastern Virginia Medical School, Norfolk, VA 23501, USA; The Leroy T. Canoles Jr. Cancer Research Center, Eastern Virginia Medical School, Norfolk, VA 23501, USA; Eastern Virginia Medical School, School of Health Professions, Norfolk, VA 23501, USA; Sentara Center for Simulation and Immersive Learning, Eastern Virginia Medical School, Norfolk, VA 23501, USA; Eastern Virginia Medical School, School of Health Professions, Norfolk, VA 23501, USA; The Leroy T. Canoles Jr. Cancer Research Center, Eastern Virginia Medical School, Norfolk, VA 23501, USA

## Abstract

**Summary:**

We present promor, a comprehensive, user-friendly R package that streamlines label-free quantification proteomics data analysis and building machine learning-based predictive models with top protein candidates.

**Availability and implementation:**

promor is freely available as an open source R package on the Comprehensive R Archive Network (CRAN) (https://CRAN.R-project.org/package=promor) and distributed under the Lesser General Public License (version 2.1 or later). Development version of promor is maintained on GitHub (https://github.com/caranathunge/promor) and additional documentation and tutorials are provided on the package website (https://caranathunge.github.io/promor/).

**Supplementary information:**

Supplementary data are available at *Bioinformatics Advances* online.

## 1 Introduction

Label-free quantification (LFQ) approaches are commonly used in mass spectrometry-based proteomics. One of the most widely used software tools for protein identification and quantification is MaxQuant (Tyanova *et al.*, 2016a). The downstream analysis of MaxQuant output files can be complex and often challenging to those inexperienced in proteomics data analysis. Some tools available for this purpose are implemented as graphical user interface (GUI) applications [e.g. LFQ-Analyst (Shah *et al.*, 2019), ProVision (Gallant *et al.*, 2020), ProteoSign (Efstathiou *et al.*, 2017)], among which, one of the most popular is the MaxQuant-associated tool, Perseus (Tyanova *et al.*, 2016b). Perseus is an extensive software suite that offers a range of features to analyze several different types of proteomics data. While Perseus is fairly easy to use, the user interface with its wide range of options can be overwhelming at times to new users. Furthermore, the inability to save previously used analytical settings in GUI applications such as Perseus may present challenges to researchers looking to standardize data analysis. Other tools, such as MSstats (Choi *et al.*, 2014), protti (Quast *et al.*, 2022), pmartR (Stratton *et al.*, 2019) and DEP (Zhang *et al.*, 2018) are primarily implemented as R packages and provide greater analytical flexibility and reproducibility to proteomics data analysis workflows. While these available software all offer analytical capability to perform the steps in typical proteomics data analysis workflows, users may need additional software to perform tasks specific to their research domain (e.g. clinical applications, biomarker discovery).

In recent years, machine learning (ML) has made its presence felt in the field of proteomics. Particularly in biomarker research, ML is becoming a popular tool to derive candidate biomarker panels from proteomics data (Bader *et al.*, 2020; Virreira Winter *et al.*, 2021). ML algorithms are now being widely employed to build proteomics-based predictive models of disease prognosis and diagnosis (Desaire *et al.*, 2022; Mann *et al.*, 2021). When building a proteomics-based predictive model, choosing a robust panel of protein candidates can greatly improve the accuracy of the model. In this regard, ML-based predictive models could benefit from narrowing down protein features to those that show significant differences in abundance between groups of interest. In the current landscape of proteomics data analytical tools, the capability to seamlessly transition from differential expression analysis to predictive modeling is limited. Realizing this need, we developed promor, a comprehensive, user-friendly, R package that streamlines differential expression analysis and predictive modeling of label-free proteomics data. promor provides an all-in-one reproducible workflow that integrates tools to perform quality control, visualization and differential expression analysis of label-free proteomics data. Furthermore, promor integrates tools to build ML-based predictive models using top protein candidates identified through differential expression analysis, assess model performance, determine feature importance and estimate the predictive power of the models.

## 2 Overview

### 2.1 Implementation

**Fig. 1.**
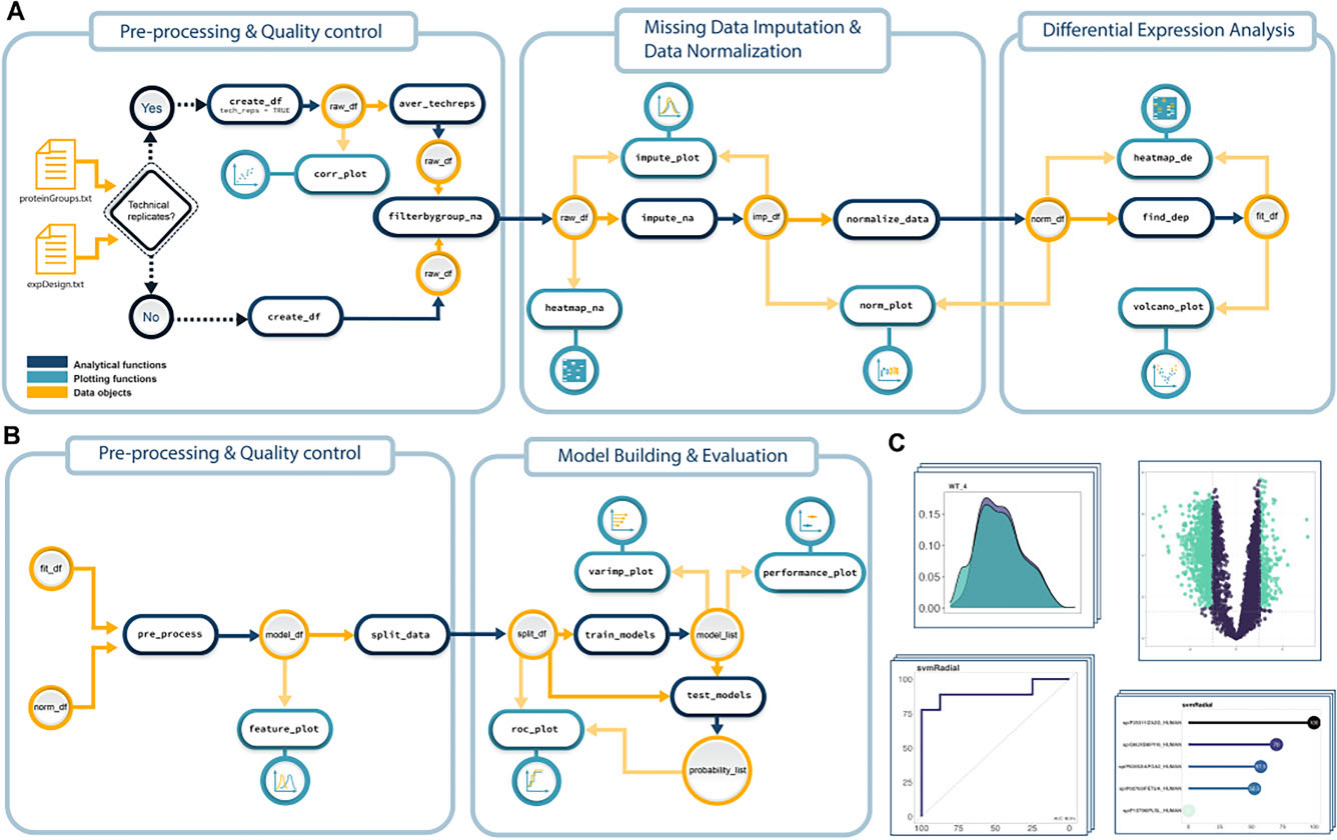
An overview of suggested promor workflows. (**A**) Proteomics data analysis workflow includes analytical functions for pre-processing, quality control, missing data imputation, data normalization and differential expression analysis. (**B**) Modeling workflow includes analytical functions for pre-processing the output of differential expression analysis, model building and model evaluation. (**C**) Several plotting functions are provided to visualize data and produce publication-ready figures using color blind-friendly palettes

promor is implemented in R (≥3.5.0) and relies on packages such as imputeLCMD (Lazar *et al.*, 2016), limma (Ritchie *et al.*, 2015) and caret (Kuhn, 2008) for back-end pre-processing, differential expression analysis and ML-based modeling, respectively. As input, promor requires a user-generated tab-delimited text file containing the experimental design and a MaxQuant-produced ‘proteinGroups.txt’ file or a standard quantitative table of protein intensities, which could be produced by any proteomic data analysis software. For visualization, promor employs the popular ggplot2 (Wickham *et al.*, 2016) architecture and produces ggplot objects, which allows for further customization (Fig. 1).

### 2.2 Proteomics data analysis

promor can be used to analyze any bottom-up label-free proteomics data (e.g. raw, LFQ or iBAQ). Multiple functions are provided for quality control, visualization, missing data imputation, normalization and differential expression analysis (Table 1 and Fig. 1A).

**Table 1.** Analytical and visualization functions in promor^a^

Function name	Input	Tasks	Output
create_df	A proteinGroups.txt file from MaxQuant/a standard input file containing a quantitative matrix of protein intensity data. A tab-delimited text file containing the experimental design.	Creates a data frame of LFQ protein intensities. Removes contaminant proteins, proteins identified only by site, reverse sequence proteins and proteins identified by two or fewer unique peptides. Converts zeros to missing values. Log_2_ transforms the values.	raw_df
aver_techreps	raw_df	If technical replicates are present in the data, computes average intensity across technical replicates for each sample.	raw_df
filterbygroup_na	raw_df	Filters out proteins with >34% missing values (<66% valid values) in at least one of the groups.	raw_df
impute_na	raw_df/norm_df	Imputes missing values using the ‘minProb’ method.	imp_df
normalize_data	raw_df/imp_df	Normalizes the data using the ‘quantile’ method.	norm_df
find_dep	norm_df/imp_df	Identifies differentially expressed proteins with an absolute log_2_ fold change >1 at an adjusted *P*-value <0.05.	fit_df
pre_process	norm_df/imp_df fit_df	Extracts protein intensity data for the top 20 differentially expressed proteins, removes proteins that show high pairwise correlation (>0.90) and converts the data into a format suitable for modeling.	model_df
split_data	model_df	Splits data into training (70%) and test (30%) data sets while preserving the overall class distribution of the data.	split_df
train_models	split_df	Trains ML models on the training data set using the default list of ML algorithms (‘svmRadial’, ‘glm’, ‘rf’, ‘xgbLinear’, ‘naive_bayes’), performs 10-fold cross validation three times, calculates re-sampling-based performance measures for the models and outputs the best model for each algorithm.	model_list
test_models	split_df model_list	Uses the models built using the training data to predict the test data.	probability_list
corr_plot	raw_df	Generates scatter plots showing the correlation between pairs of technical replicates.	ggplot
heatmap_na	raw_df	Generates a heatmap to show the missing data distribution in the matrix.	ggplot
impute_plot	raw_df/norm_df imp_df	Generates a global density plot showing the data distribution before and after missing data imputation.	ggplot
norm_plot	raw_df/imp_df norm_df	Generates box plots showing the sample data distributions before and after data normalization.	ggplot
heatmap_de	imp_df/norm_df fit_df	Generates a heatmap of protein intensities for the top 20 differentially expressed proteins.	ggplot
volcano_plot	fit_df	Generates a volcano plot highlighting significantly differentially expressed proteins (absolute log_2_ fold change >1 at an adjusted *P*-value <0.05).	ggplot
feature_plot	model_df	Generates box plots showing protein intensity differences among groups (classes).	ggplot
varimp_plot	model_list	Generates lollipop plots showing the importance of different proteins (features) in the models built.	ggplot
performance_plot	model_list	Generates boxplots showing the performance (accuracy and kappa) of models built using different ML algorithms.	ggplot
roc_plot	split_df probability_list	Generates receiver operating characteristic (ROC) curves showing the predictive power of the models built using different ML algorithms.	ggplot

a This table describes the tasks and the output produced by the functions under default settings.

To demonstrate the utility of promor for analyzing label-free proteomics data that do not contain technical replicates, we analyzed a previously published proteome benchmark data set by Cox *et al.* (2014) (PRIDE ID: PXD000279). The data set consists of LFQ protein intensity data for 6694 proteins quantified from HeLa (H) and *Escherichia coli* (L) lysates that were mixed at defined ratios. There were six samples in total. Three biological replicates represented each of the two groups. The results from the analysis were visualized at multiple stages (Supplementary Figs S1–S5). First, we pre-processed the data using the create_df function with default settings. create_df function removed contaminant proteins, proteins identified ‘only-by-site’, reverse sequence proteins and proteins identified by two or fewer unique peptides. To remove proteins with a high proportion of missing values, we used the filterbygroup_na function, setting the highest allowed missing data percentage in either group at 40%. Next, we imputed the missing data in the data frame using the impute_na function with the default ‘minProb’ method assuming that the missing values are left-censored. Since the data have already been normalized with the MaxLFQ algorithm (Cox *et al.*, 2014) in MaxQuant, we did not further normalize the data in promor. The output of imputation (imp_df object) was used in the differential expression analysis, performed using the default settings in the find_dep function. We identified 1294 significantly differentially expressed proteins between the ‘H’ and ‘L’ groups in the data (Supplementary Table S1 and Supplementary Figs S4 and S5).

Furthermore, to test the utility of promor for analyzing label-free proteomics data that contain technical replicates, we analyzed previously published data by Ramond *et al.* (2015) (PRIDE ID: PXD001584). This data set consists of LFQ protein intensity data obtained from two strains (WT—wild type and D8—ΔargP mutant) of *Francisella tularensis*, a pathogenic bacterium responsible for the zoonotic disease tularemia. The proteinGroups.txt file contained LFQ data for 1265 proteins across 18 samples representing the two conditions (WT and D8) with three biological replicates in each condition and three technical replicates for each biological replicate. A step-by-step tutorial providing a detailed description of the workflow and the implementation choices are provided here: https://caranathunge.github.io/promor/articles/promor_with_techreps.html

### 2.3 Building predictive models

In promor, multiple functions are provided to build predictive models with differentially expressed proteins and assess model performance (Table 1 and Fig. 1B). Over 200 ML algorithms are made accessible through the caret package (Kuhn, 2008) for building predictive models. For users inexperienced in complex ML algorithms, promor provides a default list of five widely used classification-based algorithms, chosen to represent a variety of ML model types (e.g. random forest, support vector machines, generalized linear models, naive bayes and gradient boosting). However, while many different algorithms can be applied to proteomics data, it is important to note that not all of them are well-suited to address the problem at hand. The choice of machine algorithms should be carefully decided according to the prediction task, data type, sample size and the number of features (proteins) in the data set.

We tested the use of promor for building predictive models by analyzing a previously published data set by Suvarna *et al.* (2021) (PRIDE ID: PXD022296). In the original study, the authors built proteomics-based classification models to predict COVID severity in patients. To avoid class imbalance in the data, only a subset of the samples were used from the original proteinGroups.txt file. The steps leading up to differential expression analysis are described in detail here: https://caranathunge.github.io/promor/articles/promor_for_modeling.html. The results from differential expression analysis (fit_df object) and the normalized data frame (norm_df object) were used in the modeling workflow. The fit_df and norm_df objects were pre-processed with the pre_process function to convert the data into a model_df object. Next, we split the data into training and test data sets using the split_data function. The training data set contained 70% of the data (29 samples), while the test data set contained the remaining 30% (6 samples). The train_models function was run on the training data set in the split_df object with four selected ML algorithms: random forest (rf), support vector machine with linear kernel (svmLinear), naive bayes (naive_bayes) and K-nearest neighbor (knn). The four algorithms were chosen based on their suitability for building models using few features (8 proteins) and samples (35 samples). Furthermore, a *k*-fold cross-validation (*k* = 10, repeats = 3) was employed to evaluate model performance. The output was used to test the models on the test data set included in the split_df object. The results from the analysis were visualized at multiple levels during the modeling workflow (Supplementary Figs. S6–S9). The model built with the ‘naive_bayes’ algorithm performed best in terms of accuracy (85.5) and Area Under the Curve (AUC = 88.9%) (Supplementary Fig. S9).

### 2.4 Benchmarking

**Fig. 2.**
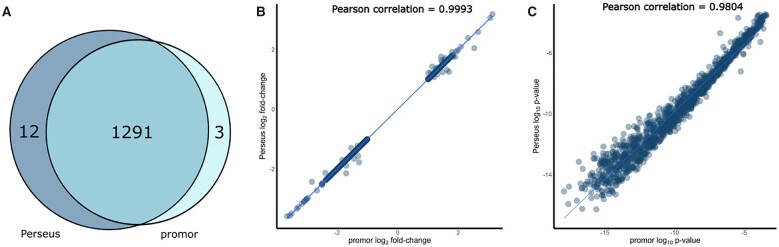
A comparison between promor and Perseus using the proteome benchmark data set, Cox *et al.* (2014). (**A**) A Venn diagram showing the overlap of the significantly differentially expressed proteins identified by promor and Perseus. Scatterplots of the resulting protein log_2_ fold changes (**B**) and log_10_*P*-values (**C**) of differentially expressed proteins as calculated by promor and Perseus

We compared the performance of promor against Perseus using the previously mentioned Cox *et al.* (2014) (PRIDE ID: PXD000279) data set. An identical workflow and parameters to those mentioned in Section 2.2 were used in Perseus. In Perseus, we used the imputeLCMD plugin to implement the ‘minProb’ imputation method, and the limma plugin to implement the moderated *t*-test. We observed a significant overlap in the differentially expressed proteins identified by both programs (98.85%) (Supplementary Tables S1 and S2 and Fig. 2A). The number of proteins that were only identified by a single program could be attributed to the random sampling during missing value imputation. Furthermore, the calculated log-fold changes and *P*-values were strongly correlated between the two programs (Fig. 2B and C). R code for benchmarking analysis is provided on github at https://github.com/caranathunge/promor_bioRxiv_preprint

## 3 Conclusions

We present promor, a user-friendly, comprehensive R package that facilitates seamless transition from differential expression analysis of label-free proteomics data to building predictive models with top protein candidates; a feature that could be particularly useful in clinical and biomarker research.

## Supplementary Material

vbad025_Supplementary_Data
