# Correction: promor: a comprehensive R package for label-free proteomics data analysis and predictive modeling

**DOI:** 10.1093/bioadv/vbad041

**Published:** 2023-04-06

This is a correction to: Chathurani Ranathunge, Sagar S. Patel, Lubna Pinky, Vanessa L. Correll, Shimin Chen, O. John Semmes, Robert K. Armstrong, C. Donald Combs, Julius O.s Nyalwidhe, promor: a comprehensive R package for label-free proteomics data analysis and predictive modeling, *Bioinformatics Advances*, Volume 3, Issue 1, 2023, vbad025, https://doi.org/10.1093/bioadv/vbad025

In the originally published version of this manuscript, in 2.2 Proteomics data analysis section, second paragraph, end of the sixth sentence should read: “[…]two or fewer unique peptides.” instead of: “[…]fewer than two peptides.”. In Table 1, column headed Tasks, first entry, ends of second sentence should read: “[…]two or fewer unique peptides.” instead of: “[…]fewer than two peptides.”.

These emendations have been made in the article.

